# Characterization of the Prostate-Specific Antigen (PSA) Catalytic Mechanism: A Pre-Steady-State and Steady-State Study

**DOI:** 10.1371/journal.pone.0102470

**Published:** 2014-07-28

**Authors:** Luigi Tomao, Diego Sbardella, Magda Gioia, Alessandra Di Masi, Stefano Marini, Paolo Ascenzi, Massimo Coletta

**Affiliations:** 1 Department of Sciences, University of Roma Tre, Roma, Italy; 2 Department of Clinical Sciences and Translational Medicine, University of Roma “Tor Vergata”, Roma, Italy; 3 Interuniversity Consortium for the Research on Chemistry of Metals in Biological Systems, Bari, Italy; 4 Interdepartmental Laboratory of Electron Microscopy, University of Roma Tre, Roma, Italy; University of Parma, Italy

## Abstract

Prostate-specific antigen (PSA), an enzyme of 30 kDa grouped in the kallikrein family is synthesized to high levels by normal and malignant prostate epithelial cells. Therefore, it is the main biomarker currently used for early diagnosis of prostate cancer. Here, presteady-state and steady-state kinetics of the PSA-catalyzed hydrolysis of the fluorogenic substrate Mu-His-Ser-Ser-Lys-Leu-Gln-AMC (spanning from pH 6.5 to pH 9.0, at 37.0°C) are reported. Steady-state kinetics display at every pH value a peculiar feature, represented by an initial “burst” phase of the fluorescence signal before steady-state conditions are taking place. This behavior, which has been already observed in other members of the kallikrein family, suggests the occurrence of a proteolytic mechanism wherefore the acylation step is faster than the deacylation process. This feature allows to detect the acyl intermediate, where the newly formed *C*-terminal carboxylic acid of the cleaved substrate forms an ester bond with the -OH group of the Ser195 catalytic residue, whereas the AMC product has been already released. Therefore, the pH-dependence of the two enzymatic steps (*i.e.*, acylation and deacylation) has been separately characterized, allowing the determination of p*K_a_* values. On this basis, possible residues are tentatively identified in PSA, which might regulate these two steps by interacting with the two portions of the substrate.

## Introduction

Prostate-specific antigen (PSA), an enzyme of 30 kDa grouped in the kallikrein family and also known as kallikrein-related peptidase 3 (KLK3) [1], is synthesized to high levels by normal and malignant prostate epithelial cells and, under pathological conditions, it is abundantly secreted in the extracellular compartments. For this reason, it is the main biomarker currently used for early diagnosis of prostate cancer. Therefore, serum levels of PSA are also useful to detect eventual recurrent forms and to follow up treatment response in not operable and metastatic tumors [2].

**Figure 1:**
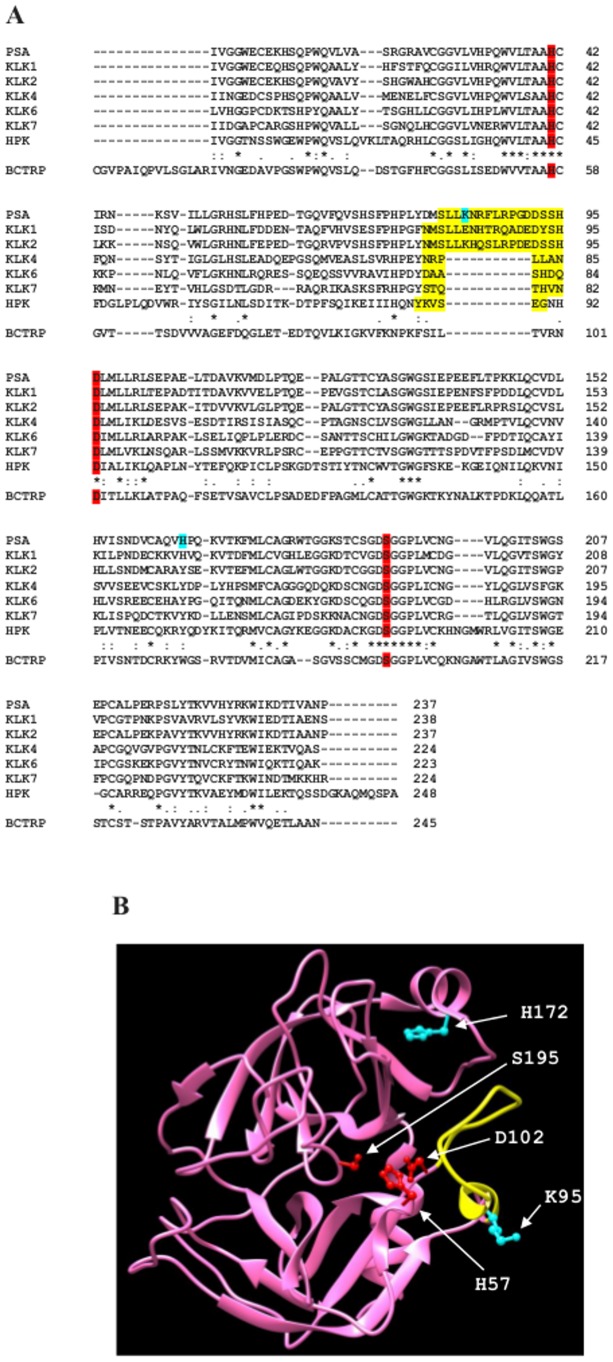
Sequence alignment of human kallikreins (panel A) and three-dimensional structure of PSA (panel B). Sequence alignment (panel A) is built with those human kallikreins for which the three-dimensional structure is available at the Protein Data Bank. The protein sequences were obtained from the NCBI database (http://www.ncbi.nlm-nih.gov). The progressive multiple alignment of PSA (also named kallikrein 3; NCBI entry number: CAD30845.1), kallikrein 1 (also named tissue kallikrein; KLK1; NCBI entry number: AAH05313.1), kallikrein 2 (KLK2; NCBI entry number: AAF08276.1), kallikrein 4 (KLK4; NCBI entry number: AAD38019.1), kallikrein 6 (KLK6; NCBI entry number: AAP35498.1), kallikrein 7 (KLK7; NCBI entry number: NP_644806.1), and human plasma kallikrein (HPK; NCBI entry number: AAF79940.1) was performed by the Clustal-Omega program (http://www.ebi.ac.uk/Tools/msa/clustalo). Only the trypsin-like serine protease domain of HPK has been aligned. The “*” symbol means that the residues are identical in all the aligned sequences; the ":" symbol indicate conserved substitutions, and the "." symbol means semi-conserved substitutions. The amino acid sequence of bovine chymotrypsinogen (BCTRP; NCBI entry number: 681083A) has been reported as the template. Three-dimensional structure of PSA (panel B). In both panels, the image was produced using UCSF Chimera molecular graphics package [26]. The “kallikrein loop” is in yellow [24], [27], [28], amino acid residues forming the catalytic triad are in red, and amino acid residues affecting the pH dependence of the catalytic parameters are in cyan.

Like all other members of the kallikrein family, PSA is a serine protease that is synthesized in an inactive form as a zymogen which is composed of a pre-peptide (also known as signal peptide) and a pro-peptide (which maintains the enzyme in the latent form). Inside the epithelial cell, the 17 amino acid pre-sequence is first cleaved off by signal peptidases. Afterwards, in the extracellular environment, the additional 7 amino acid pro-sequence is removed by human kallikrein 2 (hK2) [3]. PSA shows a conserved position of the Asp102/His57/Ser195 catalytic triad [4] (see Fig. 1). However, unlike most of kallikreins, which display a trypsin-like proteolytic specificity (*i.e.*, they cleave on the carboxyl side of a positively charged amino acid residue, namely Arg and Lys), PSA shows instead a chymotrypsin-like substrate specificity (*i.e.*, it cleaves on the carboxyl side of a hydrophobic amino acid residue, namely Tyr, Phe, Trp, and Leu). In addition, PSA is the only member of the kallikrein family that catalyzes the cleavage of substrates displaying the Gln residue at the P_1_ position [5].

Prostate cancer can increase the amount of PSA released into the blood stream, even though serum PSA is kept inactive in a variety of different forms. As a matter of fact, serum PSA falls into two general categories, namely: (*i*) free PSA, which includes all the unbound zymogen forms, and (*ii*) complexed PSA, where also active forms are kept latent through the binding of serum protease inhibitors. Notably, PSA present in the extracellular fluid, surrounding prostate epithelial cells, has been reported to be enzymatically active, suggesting that its proteolytic activity plays a role in the physiopathology of prostate cancer [6].

The most important physiological substrates for PSA have been proposed to be semenogelin I (SgI) and semenogelin II (SgII). These proteins are synthesized and secreted by the seminal vesicles in spermatic fluid and are involved in the formation of a gel matrix that wraps around ejaculated spermatozoa, preventing their functionalization (mainly via inhibition of reactive oxygen species) [7]. The gel matrix breaks down under the PSA enzymatic action, facilitating the spermatozoa movements [8]. PSA cleaves preferentially the Tyr-Glu peptide bonds and generates multiple soluble fragments of SgI and SgII [9] that seem to be the main antibacterial components in human seminal plasma [10]. These findings, together with the ability of PSA to process a number of growth regulatory proteins that are important in cancer growth and survival (such as Insulin-like growth factor binding protein, Parathyroid hormone-related protein, latent Transforming growth factor-beta 2 as well as extracellular matrix components, like fibronectin and laminin) [11]-[14], indeed suggest that PSA can facilitate tumor growth and metastasis dissemination [3], [15], [16]. On the other hand, PSA has been reported to slow down blood vessel formation, thus playing likely an important role in slowing the growth of prostate cancer [17]. As a whole, although currently PSA is a biomarker, its role in the pathobiology of prostate cancer remains obscure [3].

In view of the PSA importance both from the physiological and the pathological viewpoints, the present study is focused on insights into the catalytic mechanism of PSA. In particular, it has been investigated the PSA-catalyzed hydrolysis of the fluorogenic substrate Mu-His-Ser-Ser-Lys-Leu-Gln-AMC (Mu-HSSKLQ-AMC), a PSA-specific substrate designed on the basis of a PSA cleavage map for SgI and SgII [18]. Under pre-steady-state and steady-state conditions, the release of the Mu-HSSKLQ product (*i.e.*, the deacylation process) is the rate-limiting step of catalysis. The independent analysis of the pH dependence of the acylation and deacylation steps allows to determine the p*K*_a_ values of residues involved in the modulation of the proteolytic activity.

## Materials and Methods

PSA (pure grade >96%), obtained from seminal fluid, was purchased by SunnyLab (SCIPAC Ltd, Sittingbourne, UK). The highly-specific PSA fluorogenic substrate Mu-HSSKLQ-AMC (purity >97%) was purchased from Sigma-Aldrich (Buchs, Switzerland).

The PSA-catalyzed hydrolysis of Mu-HSSKLQ-AMC was monitored spectrofluorimetrically at 460 nm with a Cary Eclipe spectrofluorimeter (Varian, Palo Alto, Ca, USA). The excitation wavelength was 380 nm with a slit bandwidth of 5 nm. The Mu-HSSKLQ-AMC concentration ranged between 5 and 70 µM, whereas the PSA concentration was 50 nM for all determinations. The PSA-catalyzed hydrolysis of Mu-HSSKLQ-AMC was investigated between pH 6.5 and 9.0 using the following buffers: 25 mM bis-tris-HCl and 25 mM tris-HCl, in the presence of 100 mM NaCl, 10 mM CaCl_2_, and 0.05% Brij (a nonionic detergent). All measurements were performed at 37.0°C.

### Determination of kinetic parameters

The pre steady-state and steady-state parameters for the PSA-catalyzed hydrolysis of Mu-HSSKLQ-AMC were analyzed within the framework of the minimum three-step mechanism depicted by Figure 1: where E is the enzyme (*i.e.*, PSA), S is the fluorogenic peptide substrate (*i.e.*, Mu-HSSKLQ-AMC), ES is the enzyme-substrate complex, EP is the acyl intermediate, P_1_ is AMC, P_2_ is Mu-HSSKLQ, *K*_s_ is the fast pre-equilibrium constant (reflecting the actual substrate affinity for the enzyme), *k*_2_ is the acylation rate constant, and *k*_3_ is the deacylation rate constant [19].

**Figure 2:**
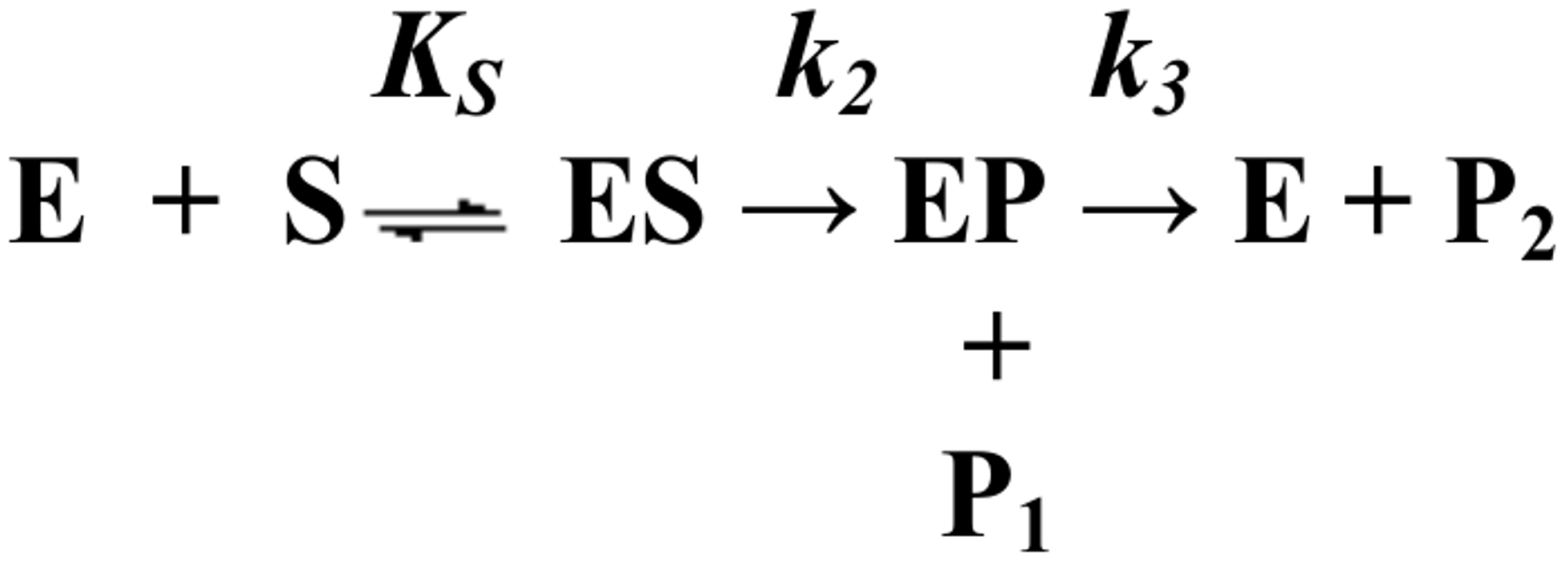
Minimum three-step mechanism underlying the pre steady-state and steady-state parameters for the PSA-catalyzed hydrolysis of Mu-HSSKLQ-AMC.



Since the fluorescence spectroscopic change is associated to the P_1_ release, the enzymatic mechanism described in Figure 2 results in a biphasic kinetic pattern whenever *k*_3_<*k*_2_ [19]. Therefore, P_1_ release has been analyzed according to Eqn 1

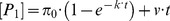
(1)where *π_0_* is the amplitude of the initial fast pre-steady-state phase (also known as the “burst”), *k* is the apparent rate constant of the initial fast pre-steady-state phase, ν indicates the subsequent slow steady-state process, and *t* is the time.

The initial fast pre-steady-state kinetics (see Eqn. 1) was analyzed according to Eqns 2 and 3 [20]: 
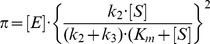
(2)and



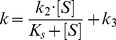
(3)The analysis of kinetics according to Eqns. (2) and (3) allowed to determine the actual concentration of active PSA (*i.e.*, [E]) and values of *K*_s_, *k*_2_, and *k*_3_.

The subsequent slow steady-state kinetics (see Eqn. 1) was analyzed according to Eqn. 4:
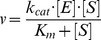
(4)where *k*_cat_ is the catalytic constant (corresponding to the rate-limiting step), *K*_m_ is the Michaelis constant, and [E] and [S] are the enzyme and substrate concentrations, respectively.

Of note, the steady-state parameters *k_cat_* and *K_m_* are related to the pre-steady-state parameters *K*_s_, *k*_2_, and *k*_3_ according to Eqns 5 and 6:
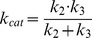
(5)


and
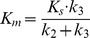
(6)


**Figure 3:**
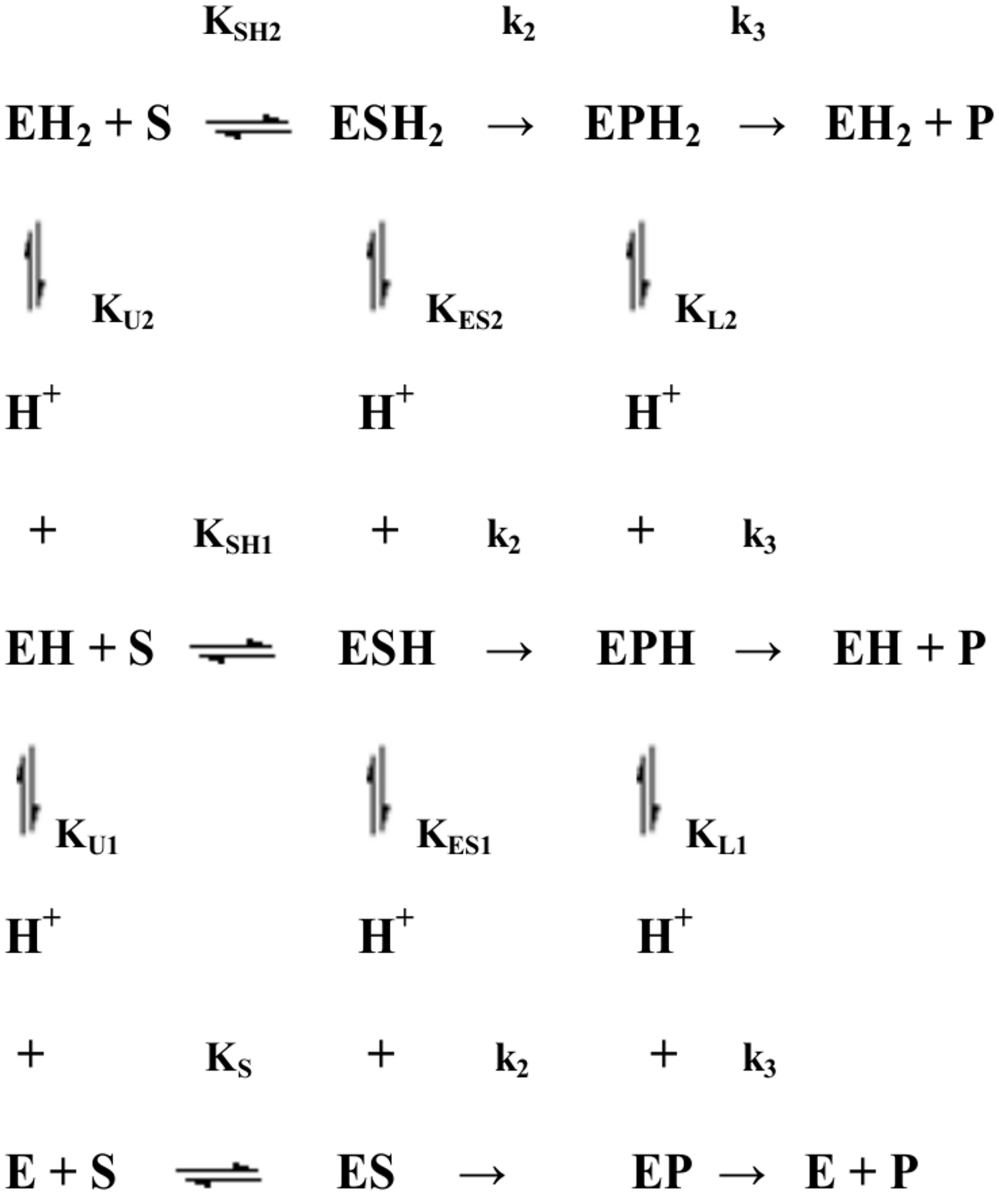
Minimum reaction mechanism for the pH dependence of pre-steady-state and steady-state parameters.



The pH dependence of pre-steady-state and steady-state parameters was analyzed in the framework of the minimum reaction mechanism depicted in Figure 3 [21], [22], where two protonating residues are involved, according to Eqns. 7-12:

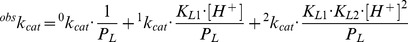
(7)

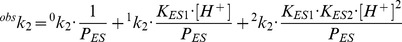
(8)




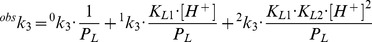
(9)




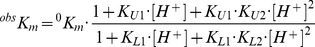
(10)




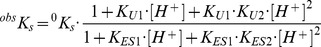
(11)




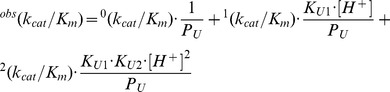
(12)where



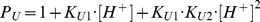
(13)

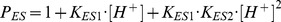
(14)




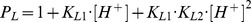
(15)


*^obs^R* refers to the observed parameter at a given pH value, *^0^R* refers to the parameter value of the unprotonated species, *^1^R* refers to the single-protonated species, and *^2^R* refers to the double-protonated species; *K_U1_* and *K_U2_* refer to the p*K_a_* values (*i.e.*, p*K_U1_* = 10*^K^* ^U1^ and p*K_U2_* = 10*^K^* ^U2^) of protonating residues in the free enzyme, *K_ES1_* and *K_ES2_* refer to the p*K_a_* values (*i.e.*, p*K_ES1_* = 10*^K^* ^ES1^ and p*K_ES2_* = 10*^K^* ^ES2^) of protonating residues in the *ES* complex and *K_L1_* and *K_L2_* refer to the p*K_a_* values (*i.e.*, p*K_L1_* = 10*^K^* ^L1^ and p*K_L2_* = 10*^K^* ^L2^) of protonating residues in the *EP* form (see Figures 1 and 2).

Kinetics of the PSA-catalyzed hydrolysis of Mu-HSSKLQ-AMC were analyzed using the MatLab program (The Math Works Inc., Natick, MA, USA). The results are given as mean values of at least four experiments plus or minus the corresponding standard deviation.

## Results and Discussion

**Figure 4:**
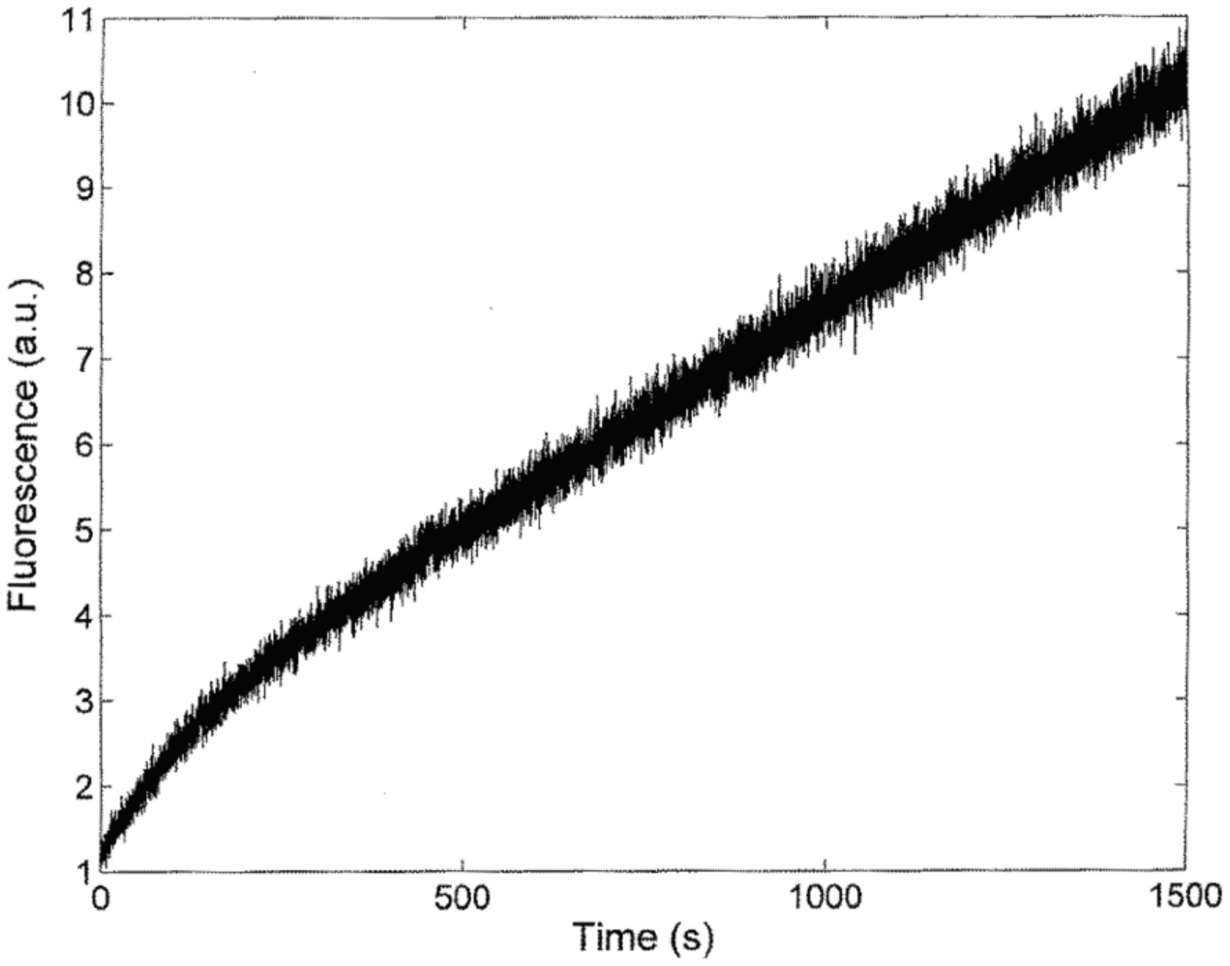
Time course of the PSA-catalyzed hydrolysis of Mu-HSSKLQ-AMC. Observation wavelength  = 460 nm, pH = 7.5 and temperature  = 37.0°C. The concentration of PSA was 50 nM. The concentration of Mu-HSSKLQ-AMC was 5 µM.

Figure 4 shows a typical time course of the PSA-catalyzed hydrolysis of the fluorogenic substrate Mu-HSSKLQ-AMC (excitation wavelength  = 380 nm; observation wavelength  = 460 nm). This kinetic pattern, observed at all pH values, is characterized by the presence of the initial “burst” phase which precedes the insurgence of the steady-state phase. This feature, which can be described by Eqn 1, has been already observed for porcine pancreatic β-kallikrein [23] and it can be referred to a mechanism where the acylation and deacylation steps of the PSA-catalyzed hydrolysis of Mu-HSSKLQ-AMC (see Fig. 2) display different rate constants [19].

**Figure 5:**
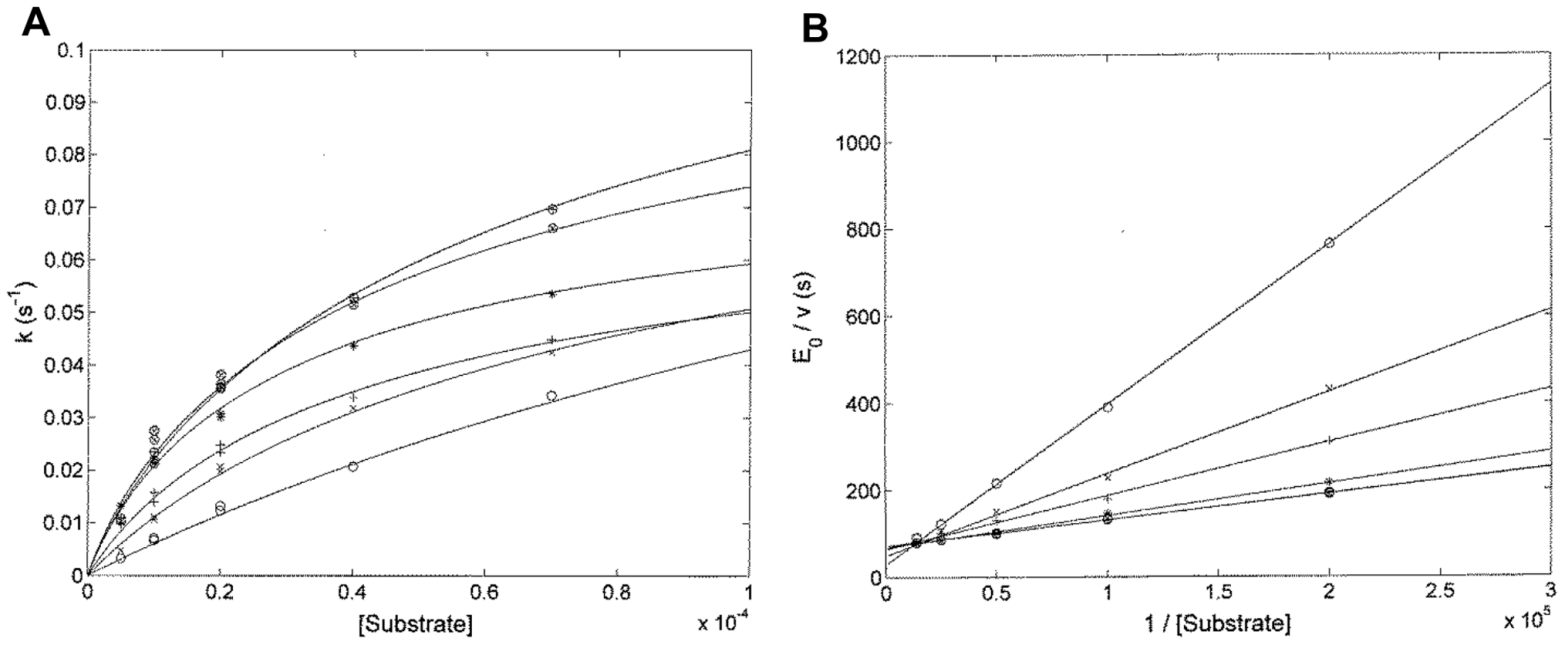
Dependence of *k* (panel A) and *v* (panel B) on the substrate concentration for the PSA-catalyzed hydrolysis of Mu-HSSKLQ-AMC. The continuous lines fitting the data reported in panels A and B were obtained according to Eqns. 3 and 4, respectively, with values of *k*_2_, *k*_3_, and *K*_s_ (panel A), and of *k*_cat_ and *K*_m_ (panel B) reported in Table 1. Values of pre-steady-state and steady-state parameters were obtained at pH 6.5 (o), pH 7.0 (x), pH 7.5 (+), pH 8.0 (*), pH 8.5 (∶), and pH 9.0 (⊕) at a temperature of 37.0°C.

Figure 5 shows the substrate concentration dependence of *k* (according to Eqn, 3, see panel A) and *v* (according to Eqn. 4, see panel B), at different pH values. Of note, the two fitting procedures are interconnected and constrained according to the relationships depicted in Eqns. 3 and 4; therefore, they are mutually consistent, resulting in the parameters reported in Table 1.

**Table 1:** Different parameters at various pH values, as obtained from the analysis of steady-state kinetics according to Eq. (1c) and of pre-steady-state kinetics according to Eq. (1d).

pH	*k_cat_* (s^−1^)	*K_m_* (M)	*k_2_* (s^−1^)	*k_3_* (s^−1^)	*K_s_* (M)
**6.5**	3.4(±0.5)×10^−2^	1.3(±0.3)×10^−4^	1.3(±0.3)×10^−1^	4.7(±0.6)×10^−2^	4.9(±0.6)×10^−4^
**7.0**	2.0(±0.3)×10^−2^	3.8(±0.5)×10^−5^	6.6(±0.9)×10^−2^	2.9(±0.5)×10^−2^	1.2(±0.3)×10^−4^
**7.5**	1.5(±0.3)×10^−2^	1.9(±0.3)×10^−5^	5.1(±0.7)×10^−2^	2.2(±0.4)×10^−2^	6.2(±0.8)×10^−5^
**8.0**	1.4(±0.3)×10^−2^	1.1(±0.2)×10^−5^	5.9(±0.9)×10^−2^	1.9(±0.3)×10^−2^	4.2(±0.7)×10^−5^
**8.5**	1.4(±0.3)×10^−2^	8.4(±1.1)×10^−6^	9.1(±1.7)×10^−2^	1.6(±0.3)×10^−2^	5.5(±0.9)×10^−5^
**9.0**	1.4(±0.2)×10^−2^	8.3(±1.0)×10^−6^	1.1(±0.2)×10^−1^	1.6(±0.3)×10^−2^	7.5(±1.0)×10^−5^

The possibility of a quantitatively satisfactory description of the two processes by parameters which are mutually consistent indeed gives a great support to the fact that the mechanism described in Figure 2 is suitable to account for the observed behavior described in Figure 4. Furthermore, the difference between *k_2_* and *k_3_* at all investigated pH values (see Table 1) indicates that the rate-limiting step is not represented by the acylation reaction of the substrate (*i.e.*, the release of AMC, as observed in many proteolytic enzymes) [20], but it resides instead in the deacylation process (*i.e.*, the release of Mu-HSSKLQ) due to the low P_2_ dissociation rate constant (*i.e.*, *k*_2_≥*k*_3_≈*k*_cat_) (see Fig. 2).

**Figure 6:**
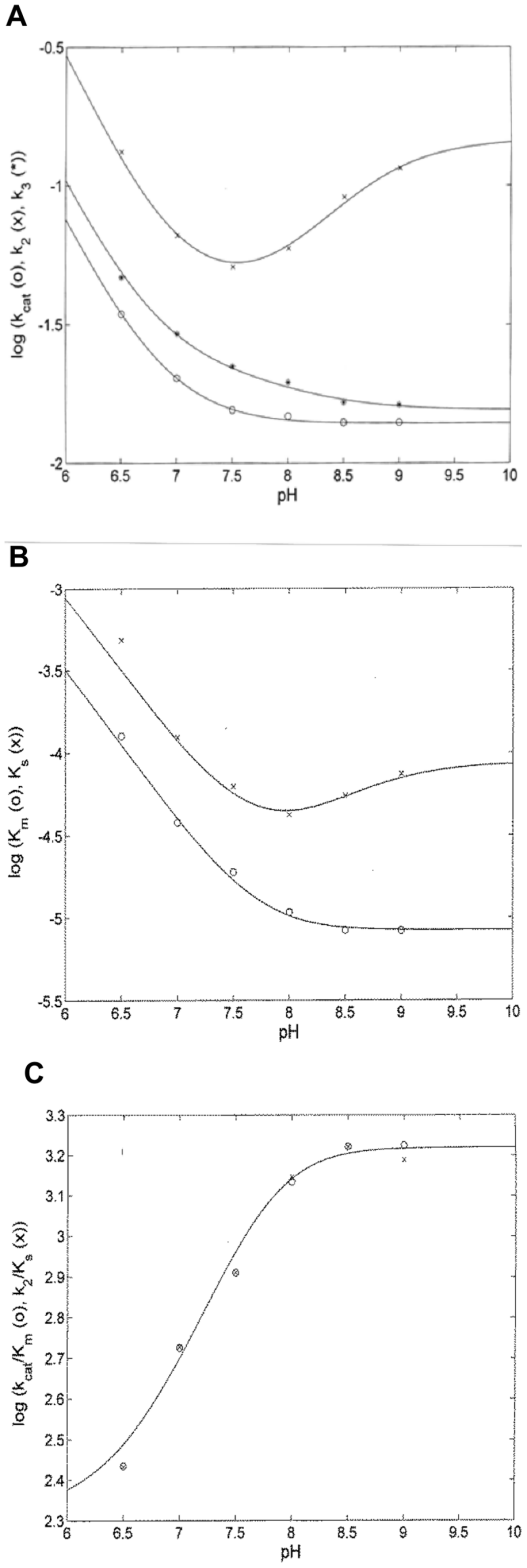
pH dependence of *k*_cat_ (o), *k*_2_ (x), and *k*_3_ (*) (panel A), of *K*_m_ (o) and *K*_s_ (x) (panel B), and of *k*_cat_/*K*_m_ (o) and *k*_2_/*K*_s_ (x) (panel C) for the PSA-catalyzed hydrolysis of Mu-HSSKLQ-AMC. The continuous lines have been obtained by non-linear least-squares fitting of data according to Eqs. 7–12 with parameters reported in Figure 6. The temperature was 37.0°C

Figure 6 shows the pH-dependence of the pre-steady-state and steady-state parameters for the PSA-catalyzed hydrolysis of Mu-HSSKLQ-AMC. The overall description of the proton linkage for the different parameters required the protonation/deprotonation of (at least) two groups with *p*K_a_ values reported in Table 2. In particular, the different *p*K_a_ values refer to either the protonation of the free enzyme (*i.e.*, E, characterized by p*K*_U1_ and p*K*_U2_; see Fig. 3) or the protonation of the enzyme-substrate complex (*i.e.*, ES, characterized by p*K*_ES1_ and p*K*_ES2_; see Fig. 3) or else the protonation of the acyl-enzyme intermediate (*i.e.*, EP, characterized by *p*K_L1_ and *p*K_L2_; see Fig. 3). The global fitting of the pH-dependence of all parameters according to Eqns. 7–12 allows to define a set of six p*K*_a_ values (*i.e.*, p*K*_U1_, p*K*_U2_, p*K*_ES1_, p*K*_ES2_, p*K*_L1_, and p*K*_L2_; see Table 2) which satisfactorily describe all proton linkages modulating the enzymatic activity of PSA and reported in Figure 3. Of note, all these parameters and the relative p*K*_a_ values are interconnected, since the protonating groups appear to modulate different parameters, which then have to display similar p*K*_a_ values, as indicated by Eqns. 7–12 (*e.g.*, p*K*_U_'s regulate *K_m_*, *K_s_* and *k_cat_*/*K_m_*, p*K*_ES_'s regulate both *K_s_* and *k_2_*, and p*K*_L_'s regulate both *K_m_*, *k_3_* and *k_cat_*); therefore, *p*K_a_ values reported in Table 2 reflect this global modulating role exerted by different protonating groups.

**Table 2:** *p*K_a_ values from the pH-dependence of various kinetic parameters.

***p*** **K_U1_**	8.02±0.16
***p*** **K_U2_**	7.61±0.18
***p*** **K_ES1_**	8.59±0.17
***p*** **K_ES2_**	5.11±0.16
***p*** **K_L1_**	8.01±0.17
***p*** **K_L2_**	5.11±0.18

**Figure 7:**
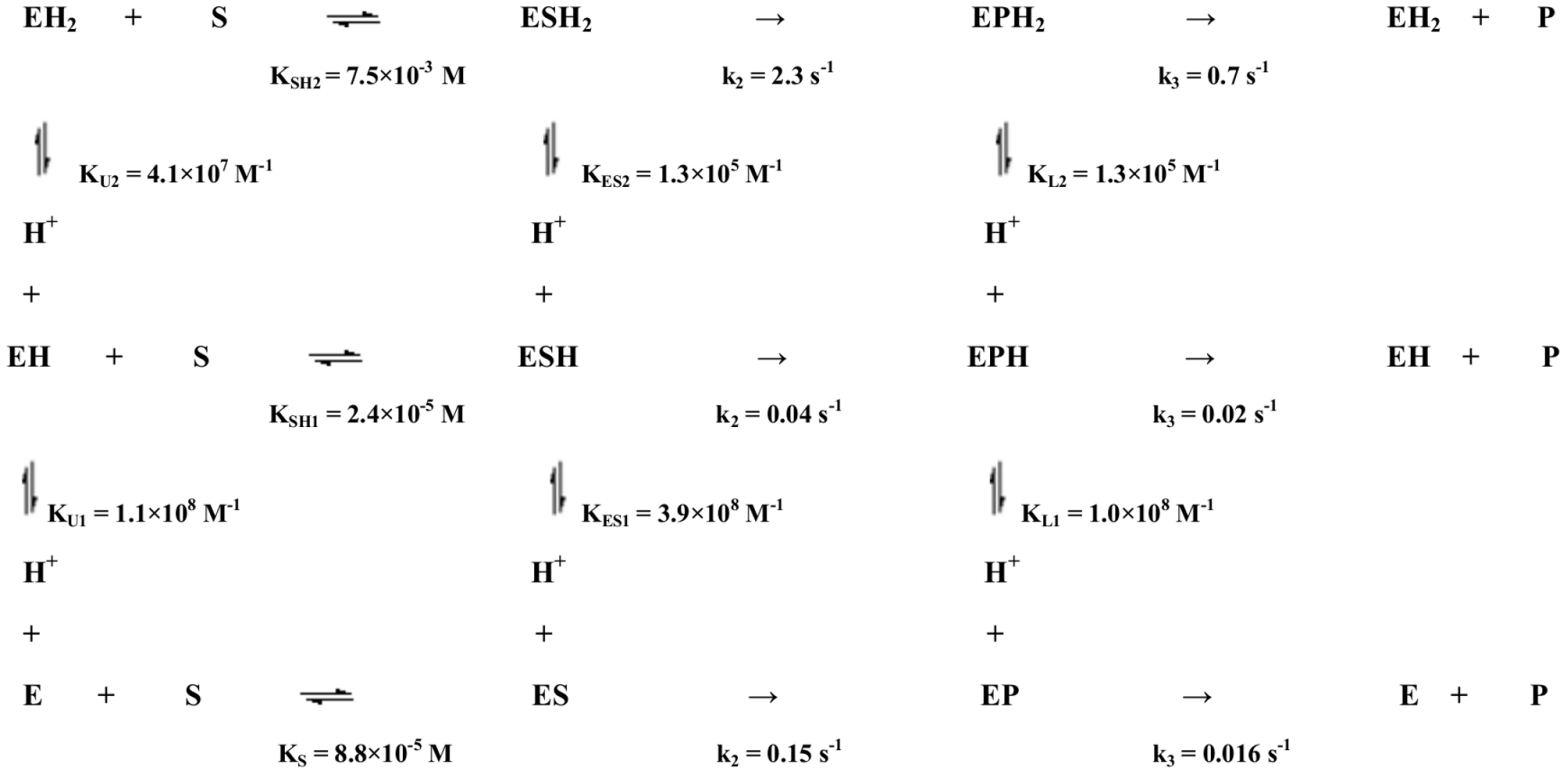
Proton-linked equilibria for the enzymatic activity of PSA at 37°C.

The inspection of parameters reported in Figure 7 envisages a complex network of interactions, such that protonation and/or deprotonation brings about modification of different catalytic parameters. In particular, the substrate affinity for the un-protonated enzyme (*i.e.*, E, expressed by *K*_S_ = 8.8×10^−5^ M; see Fig. 7) shows a four-fold increase upon protonation of a group (*i.e.*, EH, characterized by *K*_SH1_ = 2.4×10^−5^ M; see Fig. 7), displaying a p*K*_a_ = 8.0 in the free enzyme (*i.e.*, E, characterized by *K*_U1_ = 1.1×10^8^ M^−1^; see Fig. 7), which shifts to p*K*_a_ = 8.6 after substrate binding (*i.e.*, ES, characterized by *K*_ES1_ = 3.9×10^8^ M^−1^; see Fig. 7). On the other hand, this protonation process brings about a drastic five-fold reduction (from 0.15 s^−1^ to 0.036 s^−1^; see Fig. 7) of the acylation rate constant *k_2_*, which counterbalances the substrate affinity increase, ending up with a similar value of *k*_2_/*K*_S_ (or *k*_cat_/*K*_m_) over the pH range between 8.0 and 9.0 (see Fig. 6, panel C). Because of this slowing down of the acylation rate constant (*i.e.*, *k*_2_) in this single-protonated species, the difference with the deacylation rate is drastically reduced (thus *k*_2_≈*k*_3_; see Fig. 7). Further pH lowering brings about the protonation of a second functionally relevant residue, displaying a p*K*_a_ = 7.6 in the free enzyme (*i.e.*, E, characterized by *K*_U2_ = 4.1×10^7^ M^−1^; see Fig. 7), which shifts to a p*K*_a_ = 5.1 upon substrate binding (*i.e.*, *K*_ES2_ = 1.3×10^5^ M^−1^; see Fig. 7). The protonation of this residue induces a drastic 250-fold decrease of the substrate affinity for the double-protonated enzyme (*i.e.*, EH_2_, characterized by *K*_SH2_ = 7.5×10^−3^ M; see Fig. 7), even though it is accompanied by a 70-fold increase of the acylation rate constant *k*_2_ ( = 2.3 s^−1^; see Fig. 7).

The identification of these two residues, characterized by substrate-linked *p*K_a_ shifts is not obvious, even though they are likely located in the kallikrein loop [24], which is known to restrict the access of the substrate to the active site and to undergo structural readjustment(s) upon substrate binding (see Fig. 1). In particular, a possible candidate for the first protonating residue ionizing at alkaline pH is the Lys95E of the kallikrein loop [24], which might be involved in the interaction with a carbonyl oxygen, orienting the substrate; this interaction could then distort the cleavage site, slowing down the acylation rate of the ESH (see Fig.7). On the other hand, the second protonating residue ionizing around neutrality may be a histidine (possibly even the catalytic His57), whose protonation dramatically lowers the substrate affinity, though facilitating the acylation step and the cleavage process. However, this identification cannot be considered unequivocal, since additional residues might be involved in the proton-linked modulation of substrate recognition and enzymatic catalysis, as envisaged in a structural modeling study [25], according to which, beside the His57 catalytic residue, a possible role might be played also by another histidyl group, possibly His172 (according to numbering in ref. [24]) (see Fig. 1).

Interestingly, after the acylation step and the cleavage of the substrate (with dissociation of the AMC substrate fragment), the *p*K_a_ value of the first protonating residue comes back to the value observed in the free enzyme, indeed suggesting that this ionizing group is interacting with the fluorogenic portion of the substrate which has dissociated after the acylation step (*i.e.*, P_1_ in Figure 2), concomitantly to the formation of the EP complex; therefore this residue does not seem involved anymore in the interaction with the substrate, coming back to a situation similar to the free enzyme. On the other hand, the p*K*_a_ value of the second protonating residue ( = 5.1) remains unchanged after the cleavage of the substrate observed in the EP complex, indicating that this group is instead involved in the interaction with the portion of the substrate which is transiently covalently-bound to the enzyme (possibly represented by the original *N*-terminus of the peptide), the dissociation (or deacylation) of the EP adduct representing the rate-limiting step in catalysis. Therefore, for this residue, ionizing around neutrality, the transformation of ES in EP does not bring about any modification of substrate interaction with the enzyme.

As a whole, from the mechanism depicted in Figure 7 it comes out that the enzymatic activity of PSA is mainly regulated by the proton-linked behavior of two residues, characterized in the free enzyme by p*K*_U1_ = 8.0 and p*K*_U2_ = 7.6, which change their protonation values upon interaction with the substrate. The evidence emerging is that these two residues interact with two different regions of the substrate, such that (*i*) the group characterized by p*K*_U1_, which interacts with the portion released after the acylation process (probably corresponding to the original *C*-terminus of the substrate), displays a p*K*_a_ increase after substrate binding (likely reflecting the formation of an electrostatic favorable interaction in the ES complex), whereas (*ii*) the group characterized by p*K*_U2_, which interacts with the portion released after the deacylation process, displays a *p*K_a_ decrease, clearly indicating that the corresponding residue tends to be deprotonated after substrate binding. The different modulatory role of the two residues, which sense in a distinct fashion the acylating and deacylating steps, is very interesting and may represent (i) an important mechanism to regulate in macromolecular substrates the release of different proteolytic products during the catalytic function of the enzyme and (ii) a relevant aspect to design enzyme inhibitors. In this respect, it is interesting to remark that the natural occurrence of a slow deacylating step in PSA might be exploited to design new potential inhibitors. Thus, appropriate modifications of the peptide sequence might be designed, so as to indefinitely slow down the deacylation step transforming he peptide in a “suicide” inhibitor, which completely abolishes the PSA activity.
